# Comparison of Adductor Canal Block Versus Local Infiltration Analgesia on Postoperative Pain and Functional Outcome after Total Knee Arthroplasty: A Randomized Controlled Trial

**DOI:** 10.5704/MOJ.1803.002

**Published:** 2018-03

**Authors:** W Kampitak, A Tanavalee, S Ngarmukos, C Amarase, B Songthamwat, A Boonshua

**Affiliations:** Department of Anesthesiology, King Chulalongkorn Memorial Hospital, Bangkok, Thailand; ^*^Department of Orthopaedics, Chulalongkorn University, Bangkok, Thailand

**Keywords:** adductor canal block, local infiltration analgesia, pain, functional outcome, knee arthroplasty

## Abstract

**Introduction:** Total knee arthroplasty (TKA) is associated with intense postoperative pain for which effective analgesia is essential to facilitate early postoperative recovery. Adductor canal block (ACB) and local infiltration analgesia (LIA) have become increasingly involved in postoperative pain management after TKA. We aimed to compare their efficacy and outcomes in patients undergoing TKA.

**Materials and Methods:** Sixty patients undergoing unilateral TKA were randomized to receive either postoperative single-injection ACB (Group A) or LIA (Group L) during the operation. All patients received spinal anaesthesia. Primary outcome was total morphine consumption over postoperative 24 hours. Visual analog pain scale, time to first and total dosage of rescue analgesia, performance-based evaluations [timed-up and go (TUG) test, quadriceps strength], side-effects, length of hospital stay and patient satisfaction were measured.

**Results:** Fifty-seven patients were available for analysis. Median total morphine consumption over 24 and 48 postoperative hours of Group A were significantly less than Group L (6/10 mg vs 13/25 mg, p, 0.008 and 0.001, respectively). Similarly, Group A had significantly lower VAS at postoperative 6, 12 and 18 hours, VAS at ambulation on postoperative (POD) 1-3, better TUG tests on POD 2 and during POD 3 than those of Group L. However, quadriceps strength and patient satisfaction were not different between both groups.

**Conclusion:** Patients undergoing TKA with single-injection ACB required less postoperative opioids than those with LIA. Furthermore, multimodal analgesia using ACB provided better postoperative analgesia, as well as performance-based activities, than those with LIA.

## Introduction

Total knee arthroplasty (TKA) is usually associated with moderate to severe postoperative pain^1,2^. Early postoperative mobilization is critical to both reduction of immobility-related complications and achieving the optimal functional outcome following surgery. Effective postoperative analgesia, including peripheral nerve block, opioids and non-opioid medications, has been found to facilitate rehabilitation, improve patient satisfaction, and may reduce length of hospital stay^3-5^.

Femoral nerve block (FNB) may provide superior pain relief to patient-controlled analgesia (PCA) with opioids^5,6^. However, it is associated with increased risk of fall from prolonged motor blockade^7,8^. Adductor canal block (ACB) has been shown to be an alternative technique to FNB for postoperative pain control after TKA. Recent data suggested that ACB may contribute to adequate analgesia with a multimodal analgesic regimen^9-11^ and be associated with better quadriceps strength, postoperatively, in comparison with FNB^12,13^.

LIA has been shown to provide superior postoperative analgesia and earlier mobilization compared to placebo^14,15^, intrathecal morphine^16^, epidural analgesia^17,18^ and FNB^19-22^. Furthermore, LIA is less expensive and easier to perform than FNB, albeit with similar analgesic effects^23-28^. To the best of our knowledge, there has been no study comparing the head-to-head efficacy between ACB and LIA in patients undergoing TKA.

In this single-center, randomized, parallel-group, double-blinded trial, we aimed to compare the effect of ACB and LIA on established pain during postoperative period as well as ambulation ability after TKA in patients receiving spinal anaesthesia with a multimodal analgesic regimen. We hypothesized that ACB would provide similar reduction of morphine consumption during the first 24 postoperative hours (primary outcome), as well as alleviate pain during rest, movement and improve functional outcome (secondary outcomes) to those of LIA.

## Materials and Methods

**Fig. 1:**
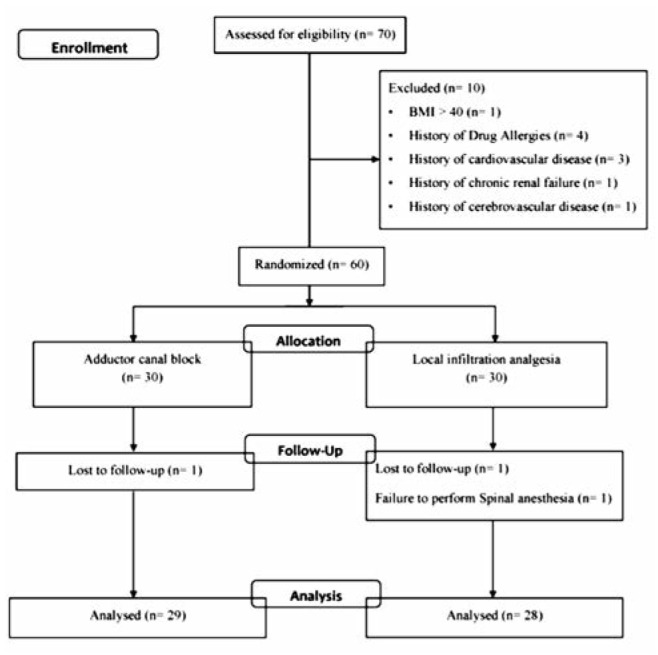
Consolidated standards of reporting trials statement flow diagram.

This study was approved by the Institutional Review Board of Chulalongkorn University, Bangkok, Thailand on March 2015 (Ref: 559/57) and registered with Clinicaltrials.in.th (TCTR20150720003). Seventy subjects scheduled to undergo elective TKA with two orthopaedic surgeons (A.T and S.N) were enrolled for this study, 60 subjects provided written consent to participate in this study and 57 subjects were available for per-protocol analysis (Fig. 1).

Eligibility criteria were primary, unilateral TKA under spinal anaesthesia, age >18 years, body mass index 18–40 kg/m2 and American Society of Anesthesiologists (ASA) functional status I–III. Exclusion criteria included contraindication for peripheral nerve or neuraxial blockade, history of allergy to drugs implicated in this study, history of abnormal liver enzymes, hepatic failure, renal insufficiency, uncontrolled hypertension, congestive heart failure, previous heart or coronary bypass surgery, history of stroke or major neurological deficit, sensory and motor disorders in the operated limb, gastritis or gastrointestinal bleeding, organ transplantation, chronic pain requiring opioid medications, neuropathic pain, failure in preoperative Timed-Up and Go (TUG) test, and subject refusal.

Demographic characteristics, preoperative VAS, functional performance-based evaluation including TUG test and quadriceps muscle strength, were recorded by a research assistant. TUG test measures the time to rise from an armchair (seat height, 46 cm), walk 3 metres, turn, and return to sitting in the same chair^29^. Quadriceps muscle strength of each subject was evaluated by a digital dynamometer [MicroFET2^TM^, Hoggan Health Industries, Salt Lake City, USA].

All patients were assigned to receive either LIA or ACB (1:1 allocation, parallel trial design), based on a computer-generated randomization (block size= 4). Group assignment was concealed via opaque envelops that were opened after enrollment. The anaesthesiologist performing ACB (W.K) was aware of the treatment, but the two surgeons (A.T and S.N) were blinded to group randomization and were not allowed to participate in postoperative patient care. Outcome assessors and clinical personnel were blinded to the study arm. The investigators were not involved in data collection.

All patients received oral acetaminophen 650 mg, 30 minutes before surgery. All surgeries were performed under spinal with paramedian approach using a 27-gauge BD^TM^ Quincke spinal needle at the L3/L4 or L2/L3 intervertebral space with the patient in the lateral position. Spinal anaesthesia using 0.5% hyperbaric bupivacaine 3 ml was used in all patients. If the spread of the sensory block was insufficient, the patient was excluded from the study, and general anaesthesia was later administered. All patients received intravenous dexamethasone 10 mg and ondansetron 4 mg for postoperative nausea and vomiting prophylaxis. The decision of whether to provide intravenous fluid during operation or to sedate using propofol was made at the discretion of the anaesthesiologist. The minimally invasive mini-midvastus approach was applied in all knees, with the use of tourniquet.

The LIA cocktail, prepared by an anaesthesiologist (W.K), was composed of 0.5% levobupivacaine 20 ml, morphine 5 mg, 1:1000 adrenaline 0.3 ml, and isotonic sodium chloride solution 40 ml. After implantation of the component and lavage of the surgical site were completed, 60 ml of LIA cocktail was injected around the prosthesis, fat and subcutaneous tissue before skin closure. In the ACB group, patients received only isotonic sodium chloride solution 60 ml for local infiltration.

We used the amount of LIA and most of mixture similar to previous study^23^ because the subjects were quite similar in weight and ethnicity to our study. Moreover, we needed to use the same dose of local anaesthetic drug to truly compare with the ACB group.

In the ACB group, ACB was performed after the surgery by a single anaesthesiologist (W.K). A high-frequency linear array ultrasound transducer [Sonosite M-Turbo, Sonosite, Bothell, Washington] was used to identify the adductor canal. The transducer was surveyed at the mid-thigh, half the distance between the inguinal crease and the patella. Next, the superficial femoral artery, the sartorius, the adductor longus and magnus muscles were identified. At this level, the adductor longus muscle could be identified underneath the sartorius muscle. The hyperechoic structure located anterolateral to the artery (saphenous nerve and nerve to vastus medialis) was identified as the target injection site. A 22-gauge, 100 mm needle [stimuplex; B Braun, Bethlehem, Pennsylvania] was introduced in-plane lateral to medial and 0.5% levobupivacine 20 ml was injected after ensuring the correct placement of the needle by using saline 2-3 ml. For the LIA group, patients received only saline 3 ml. In case of some patients wearing knee compression bandages which obscured the procedure area, temporary release at the upper part Was done.

The pain control regimen was multimodal analgesic technique. At the recovery room, all subjects were administered intravenous PCA with morphine (1mg/ml, no basal rate, 2 mg/dose, lockout interval 10 minutes, and four hours limit 30 mg) until 48 hours postoperative period. Other medications included 3 consecutive doses of intravenous parecoxib 20 mg [Dynastat, Pfizer, New York, USA] at 12-hour interval, 5 consecutive doses of oral acetaminophen 650 mg at 6-hour interval, pregabalin 75 mg [Lyrica, Pfizer, New York, USA] once daily, and celecoxib 400 mg [Celebrex, Pfizer, New York, USA], started at the last dose of parecoxib, once daily. All patients received intravenous esomeprazole 40 mg [Nexium, Astrazeneca, UK] for prevention of upper gastrointestinal bleeding, intravenous metoclopramide 5-10 mg for nausea/vomiting, intravenous chlorpheniramine 5-10 mg for itching. At discharge, home medications included half tablet of tramadol hydrochloride/acetaminophen [Ultracet, Johnson & Johnson, New Brunswick, USA] twice daily, celecoxib 200 mg, pregabalin 75 mg and esomeprazole 20 mg once a day. For severe pain, tramadol 50 mg was prescribed, orally at 6-hour interval.

Postoperative pain at rest was measured using VAS at 6, 12 and 18 hours after surgery. VAS during knee flexion and extension were measured in the morning and evening on postoperative day (POD) 1. VAS during stand-up and walking was measured on POD 2, 3. The results were recorded by research assistants who were blinded from group randomization.

Morphine consumption via PCA device was recorded at the first time requirement and 12, 24 and 48 hours, postoperatively. Quadriceps strength and TUG test on POD 2, 3 were recorded by a physiotherapist who was blinded to studied group. The incidence of nausea and vomiting (1= none, 2= queasy, 3= severe nausea, 4= vomiting), pruritus (1= none, 2= mild, 3=moderate, treatment requested, 4= severe, treatment requested), patient satisfaction (0-10), length of hospital stay, adverse events including local anesthetic toxicity and incidence of fall were recorded.

Home discharge criteria included (1) no pain on functional activities of daily living, (2) ability to get in and out of bed and a chair with little help, (3) walk along a hallway independently or with standard walker, crutches or cane, (4) ability to go up and down stairs safely. If a higher level of ongoing support was required, the patient was retained for further rehabilitation facilities.

The primary outcome was the total morphine consumption during postoperative 24 hours. Secondary outcomes included postoperative pain score, time to first and total dosage of rescue morphine in postoperative 48 hours, early and late postoperative period (from POD 0 to 3 months follow-up) performance-based test (TUG test, and quadriceps strength). Postoperative nausea and vomiting, length of hospital stay, patient satisfaction and other adverse events were also evaluated.

Sample-size calculations were done by using the morphine consumption in postoperative 24 hours as the primary endpoint. In a pilot study of 12 patients, 6 of whom received either spinal anaesthesia added to LIA or ACB. The PCA morphine consumptions were at 9± 9.2 mg and 3.7± 3.2 mg, respectively. We calculated that 27 patients would be required in each group to detect the difference with an α of 0.05 and β of 0.2. Considering the risk of dropouts, 30 patients were included in each of the two groups.

Repeated-measures analysis of variance (ANOVA) was used for the analysis of the primary outcome and secondary outcomes. Categorical data were analyzed using the Chi-square test or Fisher’s exact test. Normal distributed data were statistically tested with the independent’s t-test, and data that did not fulfill the assumptions of normal distribution were analyzed with the Mann-Whitney U-test. The results were presented as mean ± SD or median with inter-quartile range as appropriate. A p-value of <0.05 was considered statistically significant. The data was analyzed using the SPSS version 22.0 software.

## Results

There were no significant differences between groups in demographic data including age, gender, body mass index, ASA, pre-operative VAS, site of surgery, duration of surgery and length of hospital stay (Table I). Although time to first request for rescue analgesia and total median morphine consumption at the first 12 postoperative hours were not significantly different between the groups, the total median morphine consumption in Group A was significantly lower than Group L in both 24 (primary outcome) and 48 postoperative hours [6 mg (range, 0-12) vs. 13 mg (range, 5-24), p=0.008, and 10 mg (range, 4-20) vs. 25 mg (range, 12-41), p=0.001, respectively] as shown in Table II.

**Table I:** Patient characteristics

	**ACB (n=29)**	**LIA (n=28)**	**p-Value**
Age (years)	72.14±8.06	68.89 5.65	0.083
Gender			
Male	3(10.3%)	4(14.3%)	0.706
Female	26 (89.7%)	24 (85.7%)	
Height (cm)	153.59±6.91	155.52±6.72	0.289
Weight (kg)	60.94±10.45	67.38±13.95	0.053
BMI (kg/m2)	25.78±3.84	27.79±4.89	0.088
ASA			
ASA2	28 (96.6%)	28 (100%)	1
ASA3	1(3.4%)	0(0%)	
Pre-op VAS			
Rest	3.18±2.07	3.61±2.39	0.467
Movement	7.8±2.36	7.6±2.28	0.478
Surgeon			
A.T	21 (72.4%)	20 (71.4%)	0.934
S.N	8(27.6%)	8(28.6%)	
Duration of surgery (min)	120.14±25.95	130.64±28.13	0.148
Hospital stay (days)	4±0	4.11±0.31	0.083

**Table II:** Total morphine consumption (mg)

	ACB (n=29)	LIA (n=28)	p-Value
Morphine 1st time (min)	130 (105, 498)	92 (61.5, 242.5)	0.158
Morphine at 12 hr	4 (0, 8)	4 (3, 13)	0.056
Morphine at 24 hr	6 (0, 12)	13 (5, 24)	0.008*
Morphine at 48 hr	10 (4, 20)	25 (12, 41)	0.001*

^*^= significant at level 0.05

No difference in VAS was found during preoperative period between Group A and Group L (Table I). The mean VAS at 6, 12, and 18 postoperative hours in Group A were significantly lower than Group L with the differences of 1.21 (95% CI = -2.31 to -0.1, p=0.034), 1.51 (95% CI = -2.76 to - 0.27, p=0.018) and 1.4 (95% CI = -2.45 to -0.34, p=0.01), respectively.

At the first time to sit and knee extension in the morning of POD 1, the mean VAS were significantly lower in Group A than Group L (1.79±1.58 vs. 2.84±1.93, 95% CI = -1.99 to - 0.12, p=0.028; 2.24±1.65 vs. 3.61± 2.43, 95%CI = -2.47 to - 0.25, p=0.017, respectively). In the evening of POD 1, the mean VAS of knee extension in Group A was significantly lower than Group L (2.38±1.44 vs. 3.68±2.21, 95%CI = -2.3 to -0.3, p=0.012) (Table III).

**Table III:** Visual analog scale on post-operative day 1-3

	**ACB (n=29)**	**LIA (n=28)**	**p-Value**
VAS Day 1 am			
Rest	1.5 ± 1.63	2.28 ± 2.01	0.113
Sit	1.79 ± 1.58	2.84 ± 1.93	0.028*
Knee flexion	2.79 ± 1.53	3.75 ± 2.25	0.068
Knee extension	2.24 ± 1.65	3.61 ± 2.43	0.017*	
			
VAS Day 1 pm			
Rest	1.41 ± 1.28	1.7 ± 1.55	0.438
Sit	2.02 ± 1.48	2.51 ± 1.89	0.276
Knee flexion	3.18 ± 1.77	4.12 ± 2.33	0.092
Knee extension	2.38 ± 1.44	3.68 ± 2.21	0.012*
			
VAS Day 2			
Rest	1.2 ± 1.31	1.55 ± 1	0.258
Sit	1.79 ± 1.61	2.5 ± 1.29	0.072
Stand	2.38 ± 1.93	3.64 ± 1.98	0.018*
Walk	2.51 ± 1.79	3.79 ± 2.06	0.015*
			
VAS Day 3			
Rest	1.36 ± 1.36	1.24 ± 1.22	0.735
Sit	1.59 ± 1.43	2.1 ± 1.74	0.236
Stand	1.93 ± 1.38	2.93 ± 1.75	0.02*
Walk	2.21 ± 1.38	2.92 ± 1.89	0.112

^*^= significant at level 0.05

At the first time to stand and walk on POD 2, the mean VAS in Group A was significantly lower than Group L (2.38±1.93 vs. 3.64±1.98, 95%CI = -2.3 to -0.22, p= 0.018; 2.51± 1.79 vs. 3.79± 2.06, 95%CI= -2.3 to -0.26, p= 0.015, respectively) (Table III). The mean VAS on standing on POD 3 was significantly lower in Group A than Group L (1.93±1.38 vs. 2.93±1.75, 95% CI = -1.83 to -0.16, p=0.02). However, the mean VAS during walking on POD 3 was not significantly different between the groups (Table III).

**Fig. 2:**
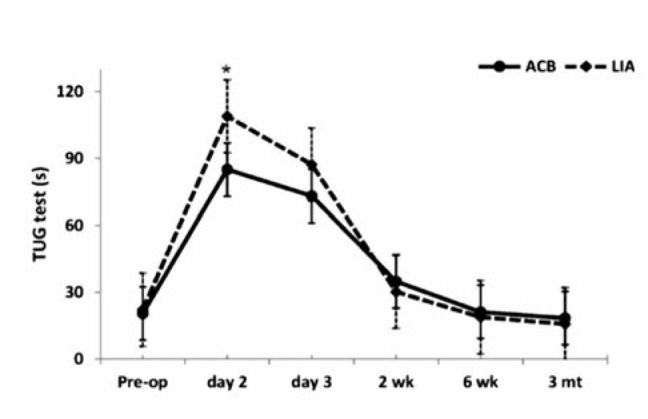
Timed-up and Go test, *= significant at level 0.05.

**Fig. 3:**
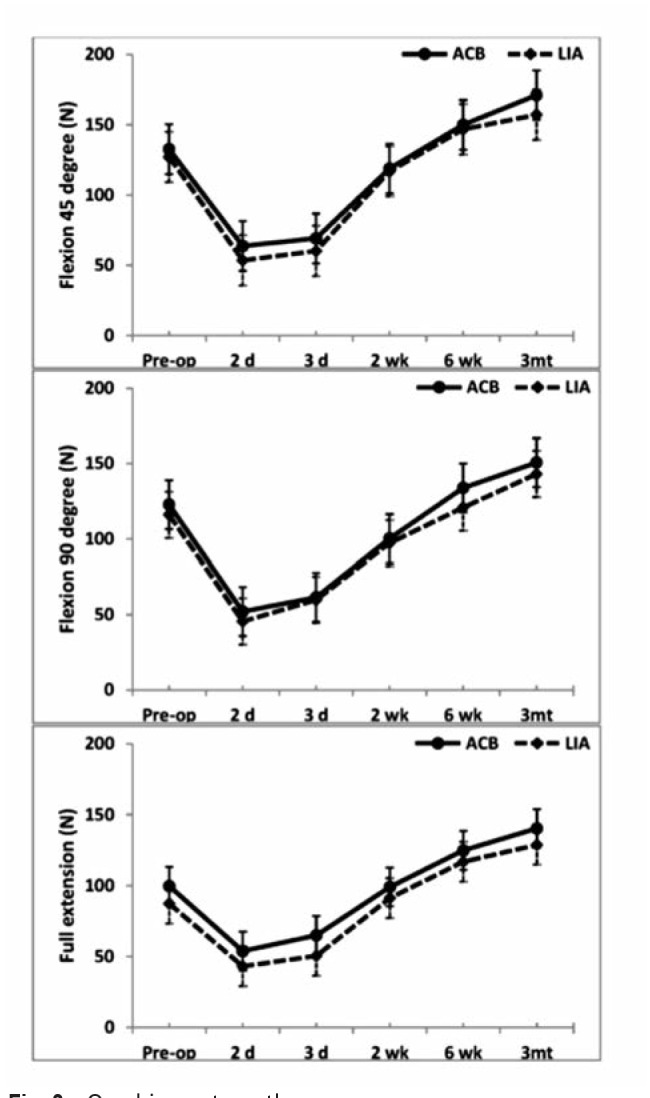
Quadriceps strength.

At preoperative period, there were no differences in TUG test and quadriceps strength on full knee extension, 45 degrees of knee flexion and 90 degrees of knee flexion between the groups (p>0.05) (Fig. 2). TUG test on POD 2 in Group A was remarkably better than Group L (mean difference= -23.95 sec, 95%CI = -42.07 to -5.83, p=0.011) (Fig. 2). Moreover, TUG test during POD 3 of Group A was significantly better than Group L (p=0.035). However, the quadriceps strengths on POD 2 and 3 of both groups were not different (p>0.05) (Fig. 3).

No differences in patient satisfaction, as well as incidence of nausea or vomiting and pruritus during postoperative period, were found between the groups (Table IV). No falls were recorded in either arm during the study period. There were no documented cases of major or minor symptoms suggestive of local anaesthetic systemic toxicity (LAST), as well as documented complications directly attributable to the nerve blocks, such as local bleeding, infection, or postoperative neuropathy.

**Table IV:** Patient satisfaction and adverse events

	**ACB (n=29)**	**LIA (n=28)**	**p-Value**
Satisfaction score	8.69 ± 1.6	8.43 ± 1.38	0.503
Fall	0	0	N/A
Nausea and vomiting			
Day 1			
none/queasy/severe nausea/vomiting	23/3/0/3	16/5/2/5	0.237
Day 2			
none/queasy/severe nausea/vomiting	24/4/0/1	21/6/0/1	0.747
Day 3			
none/queasy/severe nausea/vomiting	25/2/0/2	23/3/0/2	0.876
Pruritus			
none/mild/moderate/severe	21/5/3/0	16/9/3/0	0.406
Day 2			
none/mild/moderate/severe	20/9/0/0	19/9/0/0	0.928
Day 3			
none/mild/moderate/severe	26/3/0/0	21/7/0/0	0.179

## Discussion

In this study, under prospective, randomized, double-blind controlled trial and multimodal analgesia with comparing between ACB and LIA, we found good pain control and high satisfaction in both groups. So that both techniques can be utilised for establishing pain relief after TKA when combined with multimodal analgesic regimen especially within 12 hours postoperatively because we found no differences of morphine consumption and low pain score between the groups. However, our primary endpoint, total morphine consumption, was lower in Group A (single-shot ACB) than Group L (single-shot LIA) during both 24 and 48 hours, postoperatively. Single-shot ACB was able to provide greater pain relief than single-shot LIA during 18 hours, postoperatively. In addition, pain on movement at different times of Group A was significantly lower than Group L. In conclusion, TUG test in Group A was significantly better than Group L during 72 hours, postoperatively. These results may be considered of significant advantage since better pain relief on motion can enhance early mobilization and facilitate physiotherapy after the surgery.

First, it is likely that the nerve supply of knee sensation is more complex than expected and it may be difficult to reliably block locally after the knee is exposed^30^ and a recent study has shown better pain relief and reduced morphine consumption when addition of the ACB to LIA^31^. Second, the duration of effect of LIA may be shorter than ACB. Most previous studies had demonstrated that LIA is effective for analgesia about 6-12 hours postoperatively^32^. For the ACB, recent study^33^ showed the duration of sensory blockade of about 18-22 hours. These data were consistent with the present study in which postoperative pain score and morphine consumption in Group A were found to be less than Group L after postoperative 12 hours. Third, there were no different techniques or variability in the ACB technique as only one experienced anaesthesiologist was used in our study. Therefore, the effectiveness of the ACB may be more stable. In addition, we used the same dose of local anaesthesia, NSAIDs and other multimodal drugs in both groups to avoid areas of conflict in our study.

Our result is contradictory to a study by Sawhney *et al*^34^ who demonstrated greater pain relief at rest and movement in periarticular infiltration analgesia compared with ACB. However, the doses of local anaesthesia in both groups were not equal. They used twice the dose of local anaesthesia in periarticular infiltration analgesia which may provide better effect than ACB. Moreover, they did not exclude patients who could not receive NSAIDs by rising creatinine levels. Another study showed similar result with our study but they used different type, dose and concentration of local anesthesia between the groups^35^.

Postoperative quadriceps strength in Group A was similar to Group L, and this might be due to the low levels of postoperative pain which were observed to be similar in both groups. The motor preservation was also suggestive that ACB did not interfere with quadriceps strength, as noted in previous studies. Kwofie *et al*^13^ and Elkassabany *et al*^14^ demonstrated significant quadriceps motor sparing in ACB compared with FNB.

However, there were some limitations in our study. Although we were able to show differences in morphine consumption and pain score, a comparatively larger sample size may have helped in further reducing bias. In our study, we injected local anaesthetic drugs in proximal adductor canal near the femoral triangle. The effect of anaesthetic spread and volume may be affected more than expectation. Bendtsen *et al*^31^ applied local anaesthetic injection to the apex of femoral triangle or proximal to the adductor canal, similar to our technique, which covered more nerves that supply the knee and would control pain after TKA more than an injection inside the adductor canal. Therefore, the difference in injection site may bring about different spread of local anaesthetic drugs and may affect the duration and effectiveness of pain relief and physical outcome. Further studies would be needed to define the optimal injection site of ACB for TKA.

## Conclusion

In conclusion, a single-injection ACB with multimodal analgesia for TKA was associated with a greater reduction of morphine consumption than single-injection LIA. Furthermore, it provided superior analgesia during the postoperative 18 hours and mobilization duration. It facilitated earlier mobility after TKA than single-injection LIA. However, in clinical practice, the LIA is still easier to perform than the ACB which requires experienced anesthesiologist and may not be available in all situations.

## Conflict of Interest

The authors have no conflicts of interest to disclose.

## Funding

This work was supported by the Ratchadapiseksompotch Fund, Faculty of Medicine, Chulalongkorn University, grant number RA58/047.
